# Scientific Killing

**Published:** 1892-01

**Authors:** S. H. Preston


### SCIENTIFIC KILLING.

BY S. H. PRESTON.

It is not always certain that the doctors cure a man who survives their treatment. Some are so skeptical as to say that the man would have got well without them.

If the man dies it is "dispensation of Providence." Of course even the science of doctors must succumb to Providence.

And the doctors believe that a man had better die than be cured by any method not strictly scientific. This is the view the Empire State takes of the matter, and it makes it a crime for any but a diplomatized doctor of certain schools of medical science to cure a man. It is against the peace and dignity of the people to save life by any other school.

And still it is surmized by some that they of the scientific schools actually kill quite as many as they cure, and if a man outlives their treatment he must have a tremendous constitution. That when they take particular pains to "pull through" some celebrated patient and exhaust the skill of the entire school, they always make a botch of it, and the undertaker gets him.

Now that the State secures their services as scientific experts to kill condemned criminals, they seem to be lacking in that line. The State provides them with expensive and elaborate apparatus for the purpose, and they experiment with the most instantaneously deadly agency discovered, and they make a botch of it.

When a man accidentally touches a live electric wire he is struck dead as by lightning. When there are no supervising doctors around he dies the instant he connects with the wire. But when they set out to do a man to death by electricity it is different. They rig up their complicated chairs with all the deadly attachments of electrodes and head pieces, and switch boards that science can supply, and talk of ohms, volts, and continuous currents till they tire out each other.

But after they have strapped the poor victim in the fatal chair and given him the scientific shock that should kill him, he don't die. While they are examining him to see how dead he is, and one is pointing out to the others the proofs of *rigor mortis*, he begins to bleed and breathe and come to life again. Then adopting the adage of "If at first you don't succeed, try, try again," they turn on another killing current of electricity. But the man still bleeds and twitches and strains the straps that bind him. They try another turn of the electric current, and keep it on till the smell of burning flesh makes them sick and faint. Then they cut the wretch up to see what killed him, and come out white and trembling; sworn to secrecy of the horrors they have witnessed.

A Bowery sand-bagger could have done the business better by one blow.

